# Recurrence of Avellino Corneal Dystrophy Following Penetrating Keratoplasty: A Case Report

**DOI:** 10.31729/jnma.5726

**Published:** 2021-04-30

**Authors:** Rachana Singh Rana, Leena Bajracharya, Reeta Gurung

**Affiliations:** 1 Tilganga Institute of Ophthalmology, Gaushala, Nepal

**Keywords:** *autosomal dominant*, *granular-lattice dystrophy*, *inherited*, *keratoplasty*, *recurrence*

## Abstract

Granular - lattice (Avellino) corneal dystrophy is inherited in an autosomal dominant fashion which affects stroma of the cornea with recurrent erosions and decreased vision due to clouding of cornea in later stage. We reported a case of 53-year old woman presented with pain and blurring of vision of left eye for 10 days with history of right eye deep anterior lamellar dystrophy and Left eye penetrating keratoplasty 5years back for Avellino dystrophy. On examination right eye graft was clear and left eye showed circular edges of irregular epithelium with patchy stains and epithelial defect suggestive of recurrence of dystrophy. A patient with recurrent corneal erosions and opacity in cornea has to be examined carefully so as not to overlook Avellino corneal dystrophy. Being a rare disorder this case has been reported to draw the attention of ophthalmologists about its recurrence following keratoplasty.

## INTRODUCTION

An Avellino dystrophy is an inherited condition, autosomal dominant that affects the stromal or central layer of the cornea. It is also known as Granular-lattice corneal dystrophy. It results in the development of small granules on the cornea (granular corneal dystrophy) and lesions that resemble cracked glass (lattice corneal dystrophy). These lesions usually develop on the stromal layer before age 20 but with the growing age these become larger and more prominent involving entire stroma of cornea.^1,2^ The first case was reported in Avellino region of Italy, from which this name was derived. There is no cure for this condition and treatment usually emphasizes on diminishing symptoms, especially when vision becomes significantly impaired. Penetrating keratoplasty (PK), where a diseased cornea is entirely replaced by donated corneal tissue, can improve vision but deposits tend to recur.

## CASE REPORT

A 53-year female presented with LE Ocular pain and discomfort for 10 days. Visual acuity (VA) Right Eye (RE) uncorrected 20/80 and Left eye (LE) 20/200. It was a case of diagnosed Bilateral Avellino's dystrophy with RE Post Deep Anterior Lamellar Keratoplasty (DALK) LE Post Penetrating keratoplasty (PK) on 2015. She presented with ocular pain and discomfort on and off for 1 year and diagnosed as BL Avellino dystrophy. She undergone LE PK and after 3 months RE DALK was performed on 2015. She was on regular checkup since then. Her corneal graft was clear on both sides on last llow up and VA uncorrected RE 20/80 Refraction +1.0 Dsph/ -3.0 cylinder*90 = 20/60. LE 20/200 Refraction +1.0 Dsph/ -3.0 cylinder*150 = 20/60. There is no significant systemic history. She doesn't give family history of similar illness or other ocular disorders in the family members.

**Figure 1.**
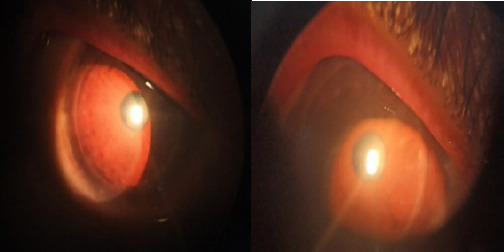
On examination-RE VA 20/80 with graft clear and No congestion and KPs.

**Figure 2.**
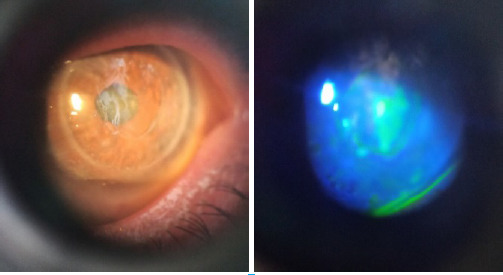
LE Post graft with irregular epithelium (A). Epithelial defect seen after Florescein stain (B).

On examination:

LE Mild congested conjunctiva. Graft in situ. Circular edges of irregular epithelium with patchy stains with epithelial defect of 1.5*1.5 mm suggestive of recurrence of dystrophy. No KP's noticed. AC formed and cells not appreciable due to hazy cornea (Figure 1, 2).

Diagnosis was made. RE Post DALK and LE Post PK with recurrence of dystrophy. It was treated with Antibiotics drops, ointment and frequent lubricants. Patient was advised to follow up after 7 days.

**Figure 3.**
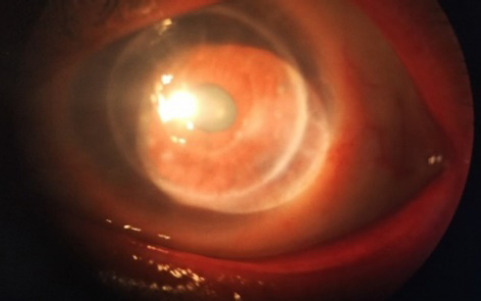
1 week follow up with no epithelial defect but hazy cornea.

On examination conjunctival congestion was less. Patient was comfortable with no epithelial defect but circular haze in the grafted cornea (Figure 3). AC was quiet and formed. Patient was counsel regarding disease progression and suggested her for close follow up.

## DISCUSSION

An Avellino dystrophy is an autosomal dominant corneal stromal disease that may have features of both granular and lattice corneal dystrophies. Molecular genetic study has shown that combined (Avellino type), granular, and lattice dystrophies share the same genetic locus on the long arm of chromosome 5 and caused by mutations in TGFB1 gene.^3,5^

It has been suggested that this combined dystrophy is due to a variation of granular dystrophy involving deposition of lattice like amyloid deposits.

This is first diagnosed in the families of Italy and named Avellino. Recent reports have described families from all around the world with this condition.

This is a non-inflammatory corneal disease that are restricted to the cornea with no systemic association and present with variable shaped corneal opacities in a clear or cloudy cornea and they affect visual acuity depending to the degree of severity.^4^

The earliest clinical evidence of ACD is the development of small, discrete and sharply demarcated granular deposits in the subepithelial and anterior stromal layers of the cornea. As the condition progresses the deposits increase in size and number, and lattice lesions develop in the mid to posterior corneal stroma.^5^

In the early stages, lesions are prevalent in the anterior central stroma, but later coalesce and occupy deeper stromal layers. Vision remains satisfactory until the fifth decade and most patients require no treatment. However, if opacities become denser and occupy the visual axis, corneal transplantation may be necessary. Most of the authors have stated that typical recurrences are superficial and early involvement is noted to be central and epithelial layer.^6^

These lattice lesions are larger, denser, whiter, and more speculated than those of lattice corneal dystrophy type I.^7^

The corneal dystrophies can be successfully treated with PK but some degree of recurrence of the original disease in the graft should be expected eventually. The order of frequency of stromal dystrophies requiring PKP and most common dystrophy to recur in a graft requiring repeat PKP was lattice followed by Avellino.^8^

In our case, patient presented with recurrent corneal erosion on and off for 1-2 years. Then keratoplasty was planned for both the eyes. Our patient with visual recovery after PK and DALK in each eye was quite significant after 4 years of keratoplasty. There is not much difference in PK and DALK as compare to visual recovery. The study done by Lyons et al.^9^ reported both surgical procedures have a good visual outcome in granular corneal dystrophy.

There is recurrence only in one eye after 4 years of transplantation which is relevant to the study done by Lyons, et al. They reported that the time of recurrence can range from 13 to 73 months and shows no significant difference between penetrating keratoplasty (PK) and lamellar keratoplasty (LK). Recurrence of the dystrophy within the graft material was almost universal within 4 years.^6^ Hence, we can conclude that counselling plays a vital role on progression and management of the disorder.

An Avellino dystrophy has rarely been reported in literature in both developing and developed countries. Although uncommon, later stage of dystrophy can result in vision loss due to clouding of cornea. We have to disseminate information about this dystrophy as this is most likely to be missed out in the early stage. The management of this disorder is symptomatic and in severe cases might need keratoplasty (PK or DALK) but recurrence can be seen after surgery. Therefore, clinicians should keep this disorder in mind while evaluating patients with recurrent corneal erosions.
